# Preventive Effect of Local Lidocaine Administration on the Formation of Traumatic Neuroma

**DOI:** 10.3390/jcm12072476

**Published:** 2023-03-24

**Authors:** Feng Ji, Yongyan Zhang, Peng Cui, Ying Li, Caixia Li, Dongping Du, Hua Xu

**Affiliations:** 1 Department of Anesthesiology, Yueyang Hospital of Integrated Traditional Chinese and Western Medicine affiliated to Shanghai University of Traditional Chinese Medicine, Shanghai 200437, China; 2 Department of Pain, Shanghai Sixth People’s Hospital affiliated to Shanghai Jiaotong University, Shanghai 200233, China

**Keywords:** traumatic neuroma, lidocaine, ultrasound guidance

## Abstract

Background: Traumatic neuroma is a common sequela of peripheral nerve injury or amputation, which often leads to severe neuropathic pain. The present study investigated the effect of local lidocaine administration on preventing the formation of traumatic neuroma. Methods: Forty-eight male Sprague–Dawley rats were randomly assigned to two groups. The lidocaine group underwent sciatic nerve transection, followed by an injection of lidocaine (0.5%) around the proximal of a severed sciatic nerve under ultrasound-guidance 2–7 days after neurectomy. In the control group, rats received an injection of saline following neurectomy. The autotomy score, mechanical allodynia, thermal hyperalgesia, histological assessment, expression of neuroma, and pain-related markers were detected. Results: Lidocaine treatment reduced the autotomy score and attenuated mechanical allodynia and thermal hyperalgesia. The mRNA expression of α-SMA, NGF, TNF-α, and IL-1β all significantly decreased in the lidocaine group in comparison to those in the saline control group. The histological results showed nerve fibers, demyelination, and collagen hyperplasia in the proximal nerve stump in the saline control group, which were significantly inhibited in the lidocaine group. Conclusions: The present study demonstrated that local lidocaine administration could inhibit the formation of painful neuroma due to traumatic nerve injury.

## 1. Introduction

Traumatic neuroma is a common sequela of peripheral nerve injury or amputation, which often leads to severe neuropathic pain. After nerve injury, the proximal end “attempts” to regenerate, the nerve fibers grow disorderly, and the regenerated axons are mixed with the proliferated collagen fibers to form a dense fibrous structure with poor vascularization, namely traumatic neuroma [1]. The formation of traumatic neuroma also changes the electrophysiological characteristics of axons. The abnormal proliferation of nerve fibers at the stump leads to the accumulation of a large number of sodium channels and spontaneous ectopic discharges, resulting in abnormal nerve conduction. This promotes the sensitivity of nerves in response to mechanical, chemical, and physical stimuli and, finally, induces neuropathic pain [2]. Traumatic neuroma is closely related to residual limb pain and phantom limb pain following amputation. Relevant studies showed that about 61% of amputated patients suffer from residual limb pain, of which 48.7% is caused by traumatic neuroma [3]. The current strategies for the prevention of traumatic neuroma include nerve stump embedment into bone marrow or adjacent muscle, nerve capping, etc. [4]. However, these surgeries are not suitable for terminal nerves, as muscles in the hand or foot are small and display, respectably, excursions during body movement, which lead to a direct traction on nerves [5]. The nerve capping technique was developed to cover the nerve stump to facilitate nerve regeneration of the severed nerve fibers. Artificial capping conduits were gradually used, but they also raised biocompatibility, swelling, and degradation rate issues [6]. Galeano et al. utilized the patient’s own femoral vein as the conduit to avoid immunogenicity [7], but this approach was not practical for clinical applications. The treatments for traumatic neuroma include analgesics, physical therapy, and surgical excision. However, the clinical efficacy of most clinical reports are still uncertain [8]. Surgical excision of the neuroma is often used clinically. The terminal nerve stump is shortened to a non-neuroma site and buried in adjacent tissue, such as bone, muscle, or a vein, but a new neuroma is prone to relapse at the new lesion site [9].

Previous studies have shown that the use of local anesthetic can prevent neuropathic pain [10,11,12]. Lidocaine is a commonly used local anesthetic. It can prevent or reduce the excessive discharge of pathologically damaged primary sensory neurons by blocking the activity of the sodium channel, so as to reduce pain [13]. Studies have shown that an intravenous drip or local administration of lidocaine can significantly reduce spontaneous and induced pain in patients with neuroma, as well as allodynia- or mechanical stimuli-induced pain [10,11,12]. When used locally, the concentration of lidocaine to block abnormal discharges of damaged nerve fibers is much lower than that to inhibit the conduction of undamaged nerves [14,15]. Local lidocaine administration of this concentration can effectively inhibit the growth of nerve axons and reduce the density of nerve fibers [16,17]. Therefore, in this study, we hypothesized that the local application of lidocaine in the early stage of nerve disconnection could inhibit nerve fiber regeneration and prevent the formation of traumatic neuroma. To verify this hypothesis, a sciatic nerve transection model was established. Repetitive injection of lidocaine was administered to the proximal end of the nerve under ultrasound guidance.

We particularly investigated the axon regeneration and perineural collagen deposition to verify its effects in the prevention of traumatic neuroma formation and release of hyperalgesia.

## 2. Materials and Methods

### 2.1. Animal and Surgical Procedures

A total of 48 adult male Sprague–Dawley (SD) rats (210–360 g) were used in the experiment. Every four rats were kept in captivity per cage, adapted to the environment controlled by temperature and humidity, with a light dark cycle of 12:12 h. The rats had free access to food and water. The rats were randomly divided into two groups: control group (*n* = 24) and lidocaine group (*n* = 24). All rats in each group were randomly divided into two subgroups (12 rats in each subgroup), and two subgroups were sacrificed at 4 weeks and 8 weeks after surgery, respectively. All animal experiments were approved by the animal ethics committee of Yueyang Hospital of Integrated Traditional Chinese and Western Medicine Affiliated to Shanghai University of Traditional Chinese Medicine (No.: YYLAC-2020-1208).

**Figure 1:**
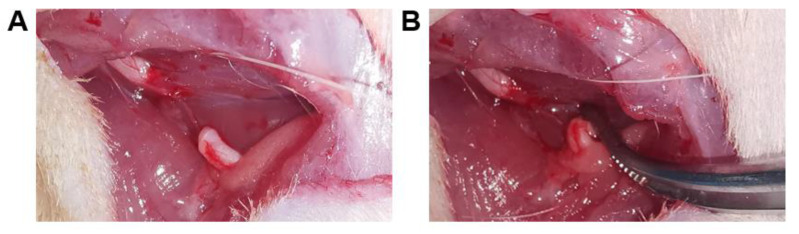
The sciatic nerve was to remove 1 cm (**A**), and the distal stump was retroflexed (**B**).

The rats were anesthetized with sevoflurane. The hair around the right thigh was shaved, and the right sciatic nerve was then exposed and transected at the center of the thigh. A 1 cm segment of the distal stump was removed and retroflexed to avoid spontaneous nerve regeneration (Figure 1). The muscle and skin were sutured layer by layer with 4-0 nylon sutures.

**Figure 2:**
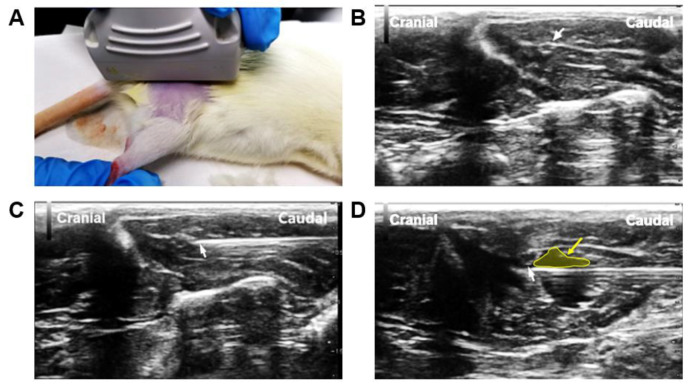
Demonstration of local lidocaine injection under the guidance of an ultrasound. (**A**) The position of the ultrasonic probe placement (L55/5–13 MHz, Hitachi noblus, Japan). (**B**) Ultrasound image of the severed sciatic nerve (the white arrow indicated the proximal end of the nerve). (**C**) Ultrasound-guided injection (the white arrow indicated the tip of the needle). (**D**) Image after lidocaine/saline injection (white arrow indicated tip of the needle; the yellow arrow and area indicated injected fluid).

An ultrasound-guided injection was performed around the proximal stump once per day from the 2nd to 7th days after surgery (Figure 2). Rats in the lidocaine group were injected with lidocaine (0.5%, 400 μL) (Hualu, China), while the saline control group used 400 μL 0.9% sodium chloride solution (Dazhong, China).

### 2.2. Behavioral Testing

Autotomy scores were recorded once a week after surgery until 8 weeks. The autotomic behavior was assessed using the modified point scale. Briefly, one point was assigned to an injury of one or more nails. Every one point was added if each distal-half toe was injured. One extra point was added for each proximal-half of an injured toe. Finally, one point was assigned for autotomy of the metatarsus and tarsal areas, respectively. An accumulated score was recorded as the final result. The maximum score for this scale was 13 points [18].

Rats were habituated to the testing environment for 3 days before testing. Paw withdrawal thresholds (PWT) were considered as mechanical allodynia. Mechanical allodynia was measured by von Frey filaments. The plantar surface of the right paw was perpendicularly subjected to a series of von Frey hairs with logarithmically incrementing stiffness until the filaments bowed slightly. Rapid paw withdrawal or flinching was considered a positive response. The 50% PWT was determined using Dixon’s up-and-down method.

Paw withdrawal latency (PWL) was considered as thermal hyperalgesia. PWL was tested using a Hargreaves radiant heat apparatus (IITC Life Science, Woodland Hills, CA, USA) with the basal paw withdrawal latency adjusted to 10 to 14 s and a cutoff of 20 s to prevent tissue damage. The stimulation was applied five times with an interval of at least 10 min.

### 2.3. Quantitative Real-Time PCR

At the end of the 4th and 8th weeks after neurotomy, the proximal segments of a severed sciatic nerve were collected from 6 rats in each group and studied by qRT-PCR for α-transcriptional changes of the α-smooth muscle actin (α-SMA), nerve growth factor (NGF), tumor necrosis factor-alpha (TNF-α), and interleukin-1β (IL-1β) genes. The total RNA was isolated with trizol reagent (Invitrogen, Carlsbad, CA, USA) and reverse transcribed into cDNA. The primers used for qPCR analysis are: α-SMA forward: 5′-GCTCCTCCAGAACGCAAATAT-3′; α-SMA reverse: 5′-GGGCCAGCTTCGTCATACTC-3′; NGF forward: 5’-CCAGTGAAATTAGGCTCCCTG-3’; NGF reverse: 5’-CCTTGGCAAAAC CTTTATTGGG-3’; TNF-α forward: 5′-CCACGCTCTTCTGTCTACTG-3′; TNF-α reverse: 5′-GCTACGGGCTTGTCACTC-3′; IL-1β reverse: 5′-CATCATCCCACGAGTCACAGAG-3′; GAPDH forward: 5′-TTCCTACCCCCAATGTATCCG-3′; and GAPDH reverse: 5′-CATGAGGTCCACCACCCTGTT-3′. The PCR test was analyzed by the ABI 7500 real-time PCR System (Applied Biosystem, Foster City, CA, USA) utilizing the SYBR^®^ Premix Ex Taq™ II Kit (TaKaRa Bio, Kusatsu, Shiga, Japan) according to the manufacturer’s instructions. GAPDH was used as an internal control. The multiple changes of the gene expression were calculated by the threshold circulation method (2−ΔΔCT).

### 2.4. Histological Evaluation

Proximal nerve segments were obtained from 6 rats in each group for the histological analysis at 4 and 8 weeks after the operation. The nerve segments were fixed with 4% paraformaldehyde and then dehydrated with ethanol, embedded in paraffin, and cut into 5 µm thick sections. The neuroma was observed by hematoxylin and eosin (H&E) staining, the nerve regeneration axon was observed by silver plating staining, the nerve myelin sheath was observed by myelin luxol fast blue (LFB) staining, and collagen hyperplasia and deposition were observed by Masson’s trichrome staining. Histological slices were observed by an optical microscope (Olympus BX50, Tokyo, Honshu, Japan).

The quantitative statistics of silver plating staining, LFB staining, and Masson’s Trichrome staining were evaluated as follows: an Eclipse Ci-L photographic microscope was used to select the target area of nerve tissue for 100× imaging. During the observation, we tried to fill the entire field of view with the tissue to ensure that the background light of each photo was consistent. The pixel area was uniformly used as the standard unit to measure the collagen area in each slice with Image-Pro Plus 6.0 analysis software. The tissue area of the corresponding field of view was recorded, and the area of nerve fibers, myelin, and collagen fibers was calculated as: percentage of nerve fibers area = nerve fibers area/total image area × 100 and percentage of myelin area = myelin area/total image area × 100. percentage of collagen fiber area = collagen fiber area/total image area × 100.

### 2.5. Statistical Analysis

The data were analyzed with GraphPad Prism 7.0 (San Diego, CA, USA) and presented as the means ± standard deviation (S.D.). A *t*-test was used to determine the significance of the differences between two groups. *p* < 0.05 was considered statistically significant.

## 3. Results

### 3.1. Assessment of Pain and Neuroma Formation

**Figure 3:**
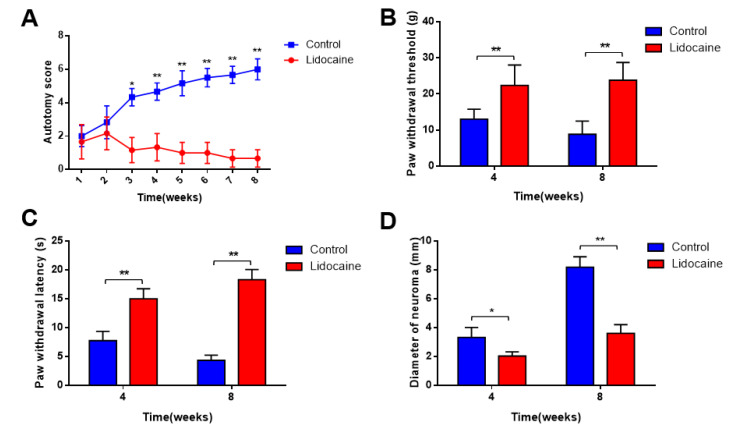
(**A**) Autotomic score in the lidocaine and saline control groups within 8 weeks after surgery. Scores in the lidocaine group were significantly lower than those in the saline control group from the 3 weeks to 8 weeks after neurectomy. Lidocaine induced a significant increase in PWT (**B**) and PWL (**C**) at 4 weeks and 8 weeks after neurectomy. (**D**) Ultrasound-guided measurement of the diameter of the proximal stump of the nerve. The maximum diameter of the proximal sciatic nerve was measured by an ultrasound. Each measurement was measured three times and averaged. The diameter was significantly thicker in the control group at 4 weeks and 8 weeks after neurectomy (*n* = 6 per group, * *p* < 0.05, ** *p* < 0.01).

The autotomic score was recorded once a week after surgery until 8 weeks. The average scores in the two groups were not significantly different during the first 2 weeks. However, from week 3 to week 8, they were significantly lower in the lidocaine group than those in the saline control group (*p* < 0.05) (Figure 3A). The formation of a neuroma after removal of the sciatic nerve may develop central sensitization and lead to hyperalgesia. To further assess the pain levels, the mechanical allodynia and thermal hyperalgesia were examined. In the saline control group, PWT and PWL decreased at 4 weeks and 8 weeks after surgery compared with the sham group (*p*  <  0.05) (Figure 3B,C). Additionally, we performed ultrasonographic diameter measurements of the proximal stump of the nerve. We noted a significant thicker diameter in the control group at 4 weeks and 8 weeks after neurectomy. The diameter of neuroma measured by an ultrasound was 3.612 ± 0.601 mm in the lidocaine group and 8.192 ± 0.781 mm in the saline control group at 8 weeks (*p* < 0.05) (Figure 3D).

**Figure 4:**
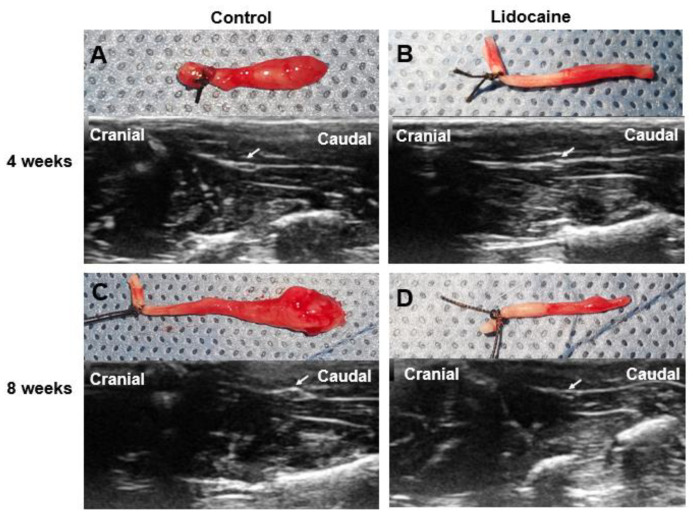
Images of the typical proximal segment of a severed sciatic nerve and its ultrasound images in two groups at 4 weeks and 8 weeks after neurectomy. A bulbous neuroma in the proximal stump was observed in the saline control group (**A**,**C**), while no obvious neuroma was seen in the lidocaine control group (**B**,**D**). The white arrow indicated the proximal end of the nerve.

At 4 weeks and 8 weeks after neurectomy, a typical bulbous neuroma could be noted in the control group, while no obvious change was found in the lidocaine group (Figure 4).

### 3.2. Expression of Neuroma and Pain-Related Markers

**Figure 5:**
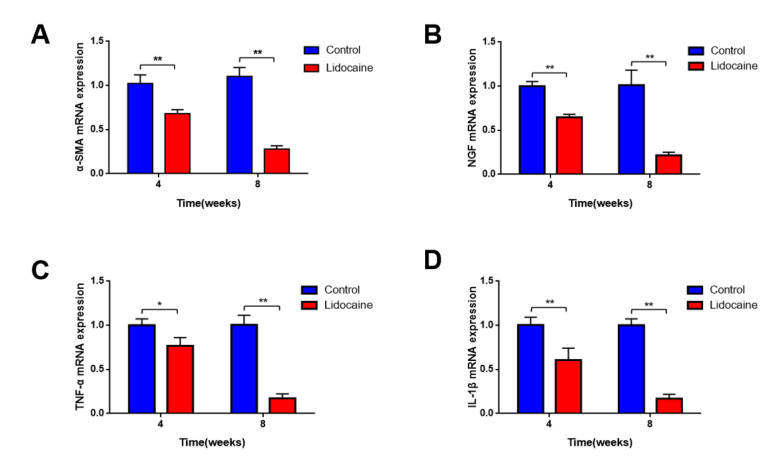
mRNA expression of α-SMA (**A**), NGF (**B**), TNF-α (**C**), and IL-1β (**D**) was significantly downregulated in the lidocaine group compared to those in the saline control group at 4 weeks and 8 weeks after neurectomy, respectively (*n* = 6, * *p* < 0.05 and ** *p* < 0.01).

Real-time quantitative PCR results showed that mRNA expression of α-smooth muscle actin (α-SMA) (Figure 5A), nerve growth factor (NGF) (Figure 5B), TNF-α (Figure 5C), and IL-1β (Figure 5D) in the proximal segments of a severed sciatic nerve was significantly downregulated in the lidocaine group when compared with the saline control group at 4 weeks and 8 weeks after neurectomy (*p* < 0.05).

### 3.3. Histological Evaluation

#### 3.3.1. Formation of Traumatic Neuroma

**Figure 6:**
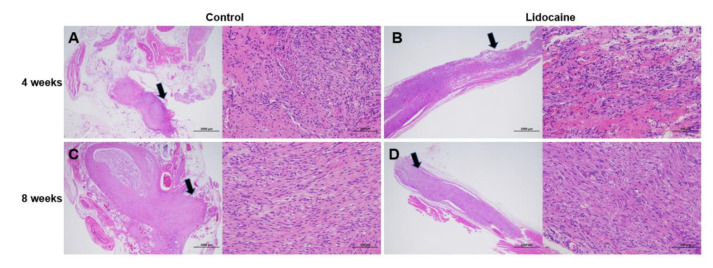
H&E staining results in the lidocaine and saline control groups at 4 weeks and 8 weeks after neurectomy. Traumatic neuroma formed in the saline control group (**A**,**C**). Observed in the lidocaine group was no obvious neuroma (**B**,**D**). Black arrows showed the position of the magnified picture.

H&E staining showed a traumatic neuroma formed at the proximal stump of the severed sciatic nerve in the saline control group. The neuroma broke through the epineurium and was composed of a haphazard proliferation of nerve fascicles, including axons and fibroblasts. The hyperplasia of Schwann’s cells was visible in the saline control group (Figure 6A,C). However, the hyperplasia of nerve fibers and Schwann’s cells was less evident, and the arrangement of nerve fibers was more orderly in the lidocaine group. No obvious traumatic neuroma was observed (Figure 6B,D).

#### 3.3.2. Nerve Fibers

**Figure 7:**
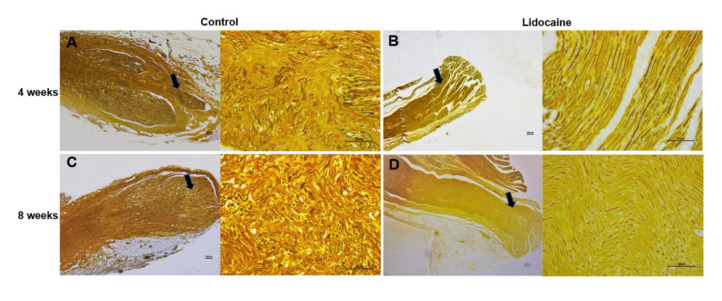

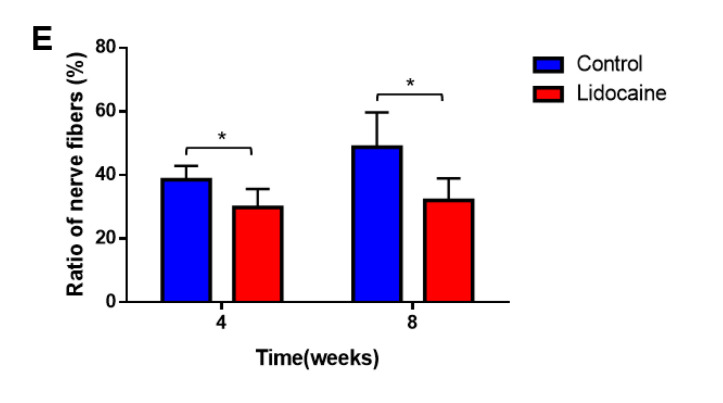
Silver plating staining showed more axons regenerated in the saline control group (**A**,**C**) than those in the lidocaine group (**B**,**D**). Quantitative results of the ratio of nerve fiber areas in the two groups at 4 weeks and 8 weeks after neurotomy (*n* = 6, * *p* < 0.05) (**E**). Black arrows showed the position of the magnified picture.

In the saline control group, silver plating staining showed that nerve fibers regenerated to spherical windings with a compact arrangement at the end of the proximal segments of a severed sciatic nerve (Figure 7A,C). However, the nerve fibers were arranged neatly in the lidocaine group (Figure 7B,D). A quantitative statistical analysis showed that the percentage of nerve fiber areas in the control group was significantly higher than that in the lidocaine group at 4 weeks (38.49 ± 1.795% vs. 29.83 ± 2.374%, *n* = 6, *p* < 0.05) and 8 weeks (48.73 ± 4.475% vs. 32.01 ± 2.843%, *n* = 6, *p* < 0.05) after neurotomy (Figure 7E).

#### 3.3.3. Demyelination

**Figure 8:**
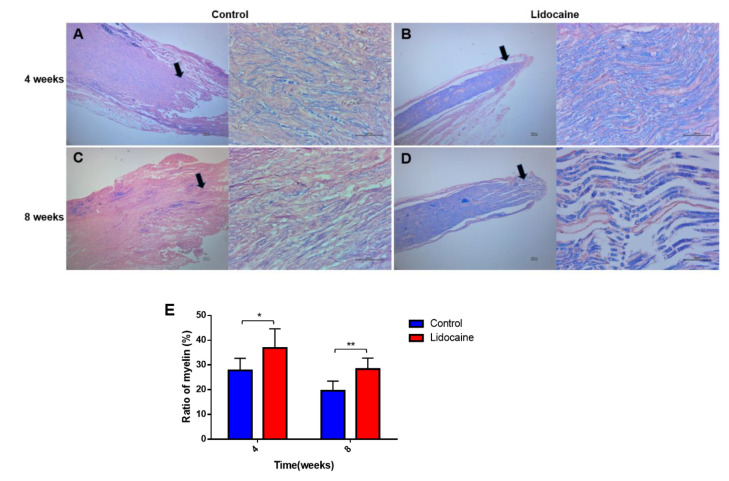
Luxol fast blue (LFB) staining showed less demyelination in the lidocaine group (**B**,**D**) than those in the saline control group (**A**,**C**). Quantitative results of the ratio of the myelin area in the two groups at 4 weeks and 8 weeks after neurotomy (*n* = 6, * *p* < 0.05 and ** *p* < 0.01) (**E**). Black arrows showed the position of the magnified picture.

LFB staining could show whether the myelin sheath was intact, degenerated, necrotic, and repaired under pathological conditions, so it was significant for both the histopathological diagnosis and research of nerves. LFB staining showed less demyelination in the lidocaine group, compared with those in the control group’s 8th week (Figure 8B,D). Myelin regeneration of the proximal segments of a severed sciatic nerve was in regular shape and thickness in the lidocaine group at the 8th week (Figure 8D). The myelin sheath of the proximal segments of a severed sciatic nerve in the saline control group was thin and disordered (Figure 8A,C). A quantitative statistical analysis showed that the percentage of myelin area decreased significantly in the control group at 4 weeks (27.76 ± 2.011% vs. 36.84 ± 3.167%, *n* = 6, *p* < 0.05) and 8 weeks (19.60 ± 1.589% vs. 28.34 ± 1.815%, *n* = 6, *p* < 0.01) after neurotomy compared with lidocaine group (Figure 8E).

#### 3.3.4. Collagen Hyperplasia and Deposition

**Figure 9:**
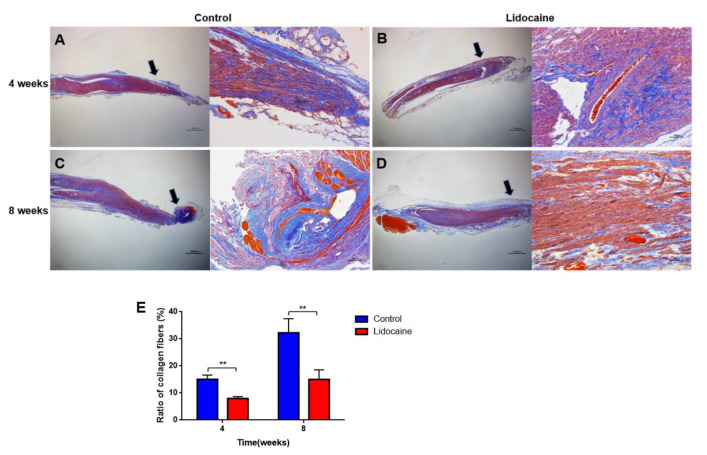
Masson’s trichrome staining in the lidocaine and saline control groups. More collagen fibers were found in the saline control group (**A**,**C**) at the 4th and 8th weeks after neurotomy, while less collagen fibers and regular distributed nerve fibers were observed in the lidocaine group (**B**,**D**). Quantitative results of the ratio of collagenous fiber areas in the two groups at 4 weeks and 8 weeks after neurotomy (*n* = 6, ** *p* < 0.01) (**E**). Black arrows showed the position of the magnified picture.

The proximal segment of the severed sciatic nerve was stained by Masson’s trichrome method in order to identify the hyperplasia of collagen at the nerve end. More collagen hyperplasia and deposition were observed in the saline control group at the 4th and 8th weeks (Figure 9A,C). Less collagen fibers and more regular distributed nerve fibers were observed in the lidocaine group instead (Figure 9B,D). The percentage of the collagen fiber area in the lidocaine group was significantly lower than that in the control group at 4 weeks (15.077 ± 1.530% vs. 7.955 ± 0.726%, *n* = 6, ** *p* < 0.01) and 8 weeks (32.288 ± 5.13% vs. 15.032 ± 3.556%, *n* = 6, ** *p* < 0.01) after neurotomy (Figure 9E).

## 4. Discussion

In the present study, we verified that a local application of low dose lidocaine would prevent the formation of traumatic neuroma and relieve neuroma-related pain. Lidocaine has been acknowledged for its analgesic and anti-hyperalgesia effects. Yatziv et al. reported that continuous administration of lidocaine to the dorsal root ganglion (DRG) with a 0.5% concentration could inhibit the discharge of ectopic impulses and reduce hyperalgesia in rats without influencing sensory or motor function [19]. In Miclescu A. et al.’s study, 1 mL of 0.1% and 0.5% lidocaine was injected close to the neuroma under ultrasound guidance to prevent spontaneous and evoked pain [20]. Both lidocaine concentrations produced a sensory loss within the area with hyperalgesia and allodynia; hypoesthesia occurred earlier and lasted longer with lidocaine 0.5% in comparison with lidocaine 0.1%. In our study, lidocaine was administered locally over a period of 5 days. Considering the neurotoxic effects of local anesthetics and the preservation of motor functions, the concentration of 0.5% lidocaine was selected. Therefore, we used 0.5% lidocaine, and similar results were also observed in our study. Significantly lower autotomic scores in the lidocaine group indicated less pain in the lidocaine-treated rats. We also observed that the autotomic scores gradually decreased since the 2nd week after neurotomy in the lidocaine group, which lasted until the 8th week. It demonstrated continuously decreased pain after ceasing of the lidocaine application.

In the local administration procedure, an ultrasound was used to ensure a precise injection of lidocaine to the proximal stump. An ultrasound is an important auxiliary tool in the diagnosis and treatment of peripheral nerve injury. It can clearly show whether the nerve is intact or partially injured. The formation of neuroma could also be easily shown as a round or oval area with low echo [21,22]. The axial resolution of our probe was 150 μm, which was much higher than that in a MR image [23]. Moreover, ultrasounds are widely used in pain management as a real-time guiding tool with high accuracy. Ultrasound-guided local injections were used for 5 consecutive days in our study. In clinical practice, high-frequency local injections may be difficult to implement. In future studies, we will improve the method of drug delivery, for example, by indwelling a drug-eluting catheter.

The histological studies in the present study explored the internal structure of neuroma and its relation to neuropathic pain. α-SMA is widely expressed in traumatic neuromas both in human and animal studies [24,25,26]. It is a marker protein for myofibroblasts, which would induce spontaneous contractions and compress nerves. Therefore, α-SMA may be associated with neuroma-induced pain [27]. Our study also showed the potential relationship between the expression of α-SMA and autotomic behavior.

The main pathological characteristics of traumatic neuroma are irregular axonal regeneration and hyperplasia or the deposition of collagen fibers. The inhibitory effect of lidocaine on the growth of axons has been confirmed in both in vivo and in vitro studies [16,17]. In the present study, glycine silver staining demonstrated a compact arrangement of axons regenerated onto the spherical windings in the proximal stump of the severed sciatic nerve in the saline control group. However, the axons were in neat alignment, and no obvious regenerated axons were observed in the lidocaine group. The mechanism may be related to the inhibition of neurite growth by the nerve growth factor (NGF) [17]. Our study also showed significantly lowered the mRNA expression of NGF in the lidocaine group than that in the saline control group. Inflammatory cytokines, such as IL-1 β, TNF- α, and IL-6, have been proven to facilitate NGF synthesis [28,29]. TNF-α and IL-1β may contribute to neuroma-associated neuropathic pain. In chronic inflammation, collagen fibers and mature myofibroblasts may infiltrate the neuroma, which are the primary source of mechanical stimulation of regenerative nerves, followed by persistent pain [30], while lidocaine could inhibit the production of these inflammatory cytokines [31,32]. The levels of IL-1β and TNF-α in the lidocaine group were also significantly lower than those in the saline control group. In this study, we found that the distribution of the collagen fibers was more structured and less dense in the lidocaine group than in the control group. Following a nerve injury, Schwann cells express higher levels of neurotrophic factors due to the need to establish a more conducive microenvironment for the regeneration, maintenance, and regulation of neuronal function [33]. We found that the expressions of NGF in the neuroma were significantly downregulated at both 4 and 8 weeks after injury in the lidocaine group as compared to the control group. Thus, lower levels of NGF may inhibit and downregulate TGF-β1, a key growth factor implicated in the promotion of collagen production, which may decrease the proliferation of scar tissue [34]. Moreover, in vitro studies have shown that lidocaine can inhibit the expression of collagen in human dermal fibroblasts, an effect that is abrogated by TGF-β1 [35]. This may be an important mechanism by which lidocaine inhibits collagen proliferation. However, the exact mechanism of this intervention is not clear yet, which requires further investigation.

There are still limitations in the study. Firstly, 8 weeks may not be enough to assess the sustained effect of lidocaine in preventing the formation of traumatic neuroma. Secondly, it is still unclear whether the concentration used in the present study is the best dose for preventing the formation of traumatic neuroma. Finally, we only evaluated the preventive effect of lidocaine on traumatic neuroma in male rats but not in female rats. In future studies, we will compare the effects of different lidocaine concentrations to explore the dose–response relationship. The effect of sex on the outcomes will also be worthwhile to explore.

## 5. Conclusions

The present study showed that local administration of 0.5% lidocaine could significantly inhibit regeneration of axons, hyperalgesia of collagen fibers, demyelination and an inflammatory response. It could finally prevent the formation of traumatic neuroma and alleviate neuropathic pain. This intervention may be rewarding for the prevention of traumatic neuroma in amputated patients.

## Data Availability

The data used to support the findings of this study are available from the corresponding author upon request.
